# COVID-19 Breakthrough Infection after Inactivated Vaccine Induced Robust Antibody Responses and Cross-Neutralization of SARS-CoV-2 Variants, but Less Immunity against Omicron

**DOI:** 10.3390/vaccines10030391

**Published:** 2022-03-03

**Authors:** Nungruthai Suntronwong, Ritthideach Yorsaeng, Jiratchaya Puenpa, Chompoonut Auphimai, Thanunrat Thongmee, Preeyaporn Vichaiwattana, Sitthichai Kanokudom, Thaneeya Duangchinda, Warangkana Chantima, Pattarakul Pakchotanon, Suvichada Assawakosri, Pornjarim Nilyanimit, Sirapa Klinfueng, Lakkhana Wongsrisang, Donchida Srimuan, Thaksaporn Thatsanatorn, Natthinee Sudhinaraset, Nasamon Wanlapakorn, Yong Poovorawan

**Affiliations:** 1 Center of Excellence in Clinical Virology, Faculty of Medicine, Chulalongkorn University, Bangkok 10330, Thailand; suntronwong.n@gmail.com (N.S.); ritthideach.yor@gmail.com (R.Y.); jiratchaya.pu@gmail.com (J.P.); chompoonut.bit@gmail.com (C.A.); tata033@hotmail.com (T.T.); preeya_teiy@hotmail.com (P.V.); kanokudom_s@yahoo.com (S.K.); suvichada.assawa@gmail.com (S.A.); mim_bhni@hotmail.com (P.N.); sirapa.klinfueng@gmail.com (S.K.); Lakkhana4118@gmail.com (L.W.); donchida.s@gmail.com (D.S.); thaksapohnl@hotmail.com (T.T.); dr_natthinee@hotmail.com (N.S.); nasamon.w@chula.ac.th (N.W.); 2 Molecular Biology of Dengue and Flaviviruses Research Team, National Center for Genetic Engineering and Biotechnology (BIOTEC), National Science and Development Agency, NSTDA, Pathum Thani 12120, Thailand; Thaneeya.dua@biotec.or.th (T.D.); mk_pk@msn.com (P.P.); 3 Division of Dengue Hemorrhagic Fever Research, Faculty of Medicine, Siriraj Hospital, Mahidol University, Bangkok 10700, Thailand; Warangkana_ch1@hotmail.com; 4 Siriraj Center of Research Excellence in Dengue and Emerging Pathogens, Faculty of Medicine, Siriraj Hospital, Mahidol University, Bangkok 10700, Thailand; 5 Royal Society of Thailand (FRS(T)), Sanam Sueapa, Dusit, Bangkok 10330, Thailand

**Keywords:** breakthrough, infection, SARS-CoV-2, omicron, inactivated virus

## Abstract

The emergence of severe acute respiratory syndrome coronavirus 2 (SARS-CoV-2) variants and the waning of immunity in vaccinated individuals is resulting in increased numbers of SARS-CoV-2 breakthrough infections. This study investigated binding antibody responses and neutralizing activities against SARS-CoV-2 variants, in patients with COVID-19 who had been fully vaccinated with CoronaVac (*n* = 77), individuals who had been fully vaccinated with CoronaVac but had not contracted COVID-19 (*n* = 170), and individuals who had received AZD1222 as a third vaccination (*n* = 210). Breakthrough infection was generally detected approximately 88 days after the second CoronaVac vaccination (interquartile range 68–100 days). Blood samples were collected at a median of 34 days after infection. Binding antibody levels in sera from patients with breakthrough infection were significantly higher than those in individuals who had received AZD1222 as a third vaccination. However, neutralizing activities against wild-type and variants, including alpha (B.1.1.7), beta (B.1.351), and delta (B.1.617.2), were comparable in patients with breakthrough infections and individuals who received a third vaccination with AZD1222, which exceeds 90%. Omicron (B.1.1.529) was neutralized less effectively by serum from breakthrough infection patients, with a 6.3-fold reduction compared to delta variants. The study suggests that breakthrough infection after two doses of an inactivated vaccine can induce neutralizing antibodies against omicron. Further investigation is needed to assess the long-term persistence of antibodies against the omicron variant.

## 1. Introduction

Since the commencement of the coronavirus disease 2019 (COVID-19) outbreak at the end of 2019, there have been more than 313 million cases of infection [1]. Vaccines are an available tool to prevent and control this threat. In Thailand, the CoronaVac vaccine was approved for Thai adults, aged 18–59, and a mass vaccination campaign was started in late February 2021 [2]. The CoronaVac vaccine is an inactivated virus vaccine administered in a two-dose schedule, 21 to 28 weeks apart [3]. Although the AZD1222 was approved in the following month for adults ≥18 years, this vaccine was prioritized for the elderly, ≥60 years of age, and individuals who had underlying diseases due to a shortage in vaccine supply. AZD1222 is Chimpanzee adenovirus Oxford 1 (ChAdOx1)-vectored vaccine, containing severe acute respiratory syndrome coronavirus 2 (SARS-CoV-2) spike protein [4]. The vaccine is given as a series of two doses, with 10 weeks intervals. However, reduced viral susceptibility to vaccine-induced antibodies due to the emergence of SARS-CoV-2 variants, together with the waning of vaccine-induced immunity, is increasing the incidence of COVID-19 breakthrough infection after vaccination [5,6]. In Thailand, implementing a third dose booster with AZD1222, following the two doses of CoronaVac, has been recommended since June 2021 [7]. However, the quick spread of new emerging omicron variants has raised concern about whether the pre-existing immunity could neutralize this variant. Several studies indicated that the neutralizing antibody titres against omicron increase after boosting with the third dose of viral vectored vaccine or mRNA vaccine [8,9,10,11]. Notably, however, the study of immunogenicity and neutralization of SARS-CoV-2 variants, particularly omicron (B.1.1.529), is limited [8]. 

This study aims to determine antibody levels and cross-neutralization in patients with SARS-CoV-2 breakthrough infection, following two doses of CoronaVac, compared with those in uninfected individuals who received two doses of CoronaVac, and those who received AZD1222 as a third vaccination.

## 2. Materials and Methods

### 2.1. Study Participants

Patients who had been completely vaccinated with two doses of CoronaVac then subsequently became infected with SARS-CoV-2 as determined via a positive polymerase chain reaction (PCR) test were recruited at the Center of Excellence in Clinical Virology between 21 April and 20 September 2021. The SARS-CoV-2 variants circulating in Thailand during the study period were alpha (April to June) and delta (July to September) (Figure S1). Controls were unexposed SARS-CoV-2 individuals who had been fully vaccinated with two doses of CoronaVac and screened with anti-nucleocapsid IgG as previously described [7]. Fully vaccinated CoronaVac individuals who received AZD1222 as a third vaccination were also analysed [7]. The study protocol was conducted in accordance with the Declaration of Helsinki and Good Clinical Practice principles, and it was approved by the Research Ethics Committee of the Faculty of Medicine, Chulalongkorn University (IRB numbers 835/64). All participants provided written informed consent.

### 2.2. Binding—Antibody and Surrogate Virus Neutralization Test (sVNT)

All sera were tested for SARS-CoV-2-specific binding antibody responses via enzyme-linked immunosorbent assays, including total immunoglobulin (Ig) against the SARS-CoV-2 receptor-binding domain (RBD), anti-RBD IgG and anti-N IgG [7]. Total RBD Ig level with value ≥0.8 U/mL was considered positive (Roche Diagnostics, Basel, Switzerland). The positive level of anti-RBD IgG is considered as value ≥50 arbitrary unit (AU)/mL or ≥7.1 binding antibody unit (BAU)/mL (multiplying AU/mL by 0.142 to convert it to BAU/mL). Additionally, the anti-N IgG with a value ≥1.4 was defined as positive (Abbott Diagnostics, Abbott Park, IL, USA). We determined the neutralizing activity using surrogate virus neutralization test (sVNT) based on antibody-mediated blockage of the interaction between the viral receptor-binding domain (RBD) and angiotensin-converting enzyme 2 (ACE2) protein [12]. A subset of samples was tested against the recombinant RBD from wild-type SARS-CoV-2 (Wuhan) and variants including alpha (B.1.1.7; N501Y), beta (B.1.351; K417N, E484K, and N501Y), and delta (B.1.617.2; L452R and T478K) assessed using a cPass^TM^ SAR-CoV-2 neutralizing antibody detection kit (GenScript, Piscataway, NJ, USA) in accordance with the manufacturer’s instructions. Briefly, serially diluted serum samples (1:10 with sample dilution buffer) were pre-incubated with horseradish peroxidase-conjugated recombinant SARS-CoV-2 RBD protein for 30 min at 37 °C. The mixture of sera-RBD was added onto human-angiotensin-converting enzyme 2 protein-coated ELISA plates for 15 min at 37 °C. After washing three times, the TMB substrate was added to develop the chromogenic reaction. After incubation, the stop solution was added to quench the reaction. The absorbance was measured immediately at 450 nm. Percentage of inhibition was calculated as inhibition (%) = (1: OD value of sample/average OD of negative control) × 100. A value ≥30% scored positive, indicating the presence of neutralizing antibodies.

### 2.3. Focus Reduction Neutralization Test (FRNT50)

Live SARS-CoV-2 neutralizing antibody titres were performed using serum from breakthrough infection and measured using a 50% focus reduction neutralization test (FRNT50) against isolates of the delta (B.1.617.2; accession number: EPI_ISL_8547018) and omicron (B.1.1.529; accession number: EPI_ISL_8547017) variants. Briefly, serum from patients with breakthrough infection was heated-inactivated at 56 °C for 30 min and performed serial dilutions spanning a range from 1:10 to 1:7290. The diluted sea was mixed with the virus (delta or omicron) and incubated at 37 °C for 1 hour. The virus-sera mixtures were added to monolayers of Vero cells in a 96-well plate and incubated for 2 hours. Then, 1.5% carboxymethyl cellulose overlay was added and incubated at 37 °C. Plates were incubated with anti-NP human mAb and then followed by peroxidase-conjugate goat anti-human IgG. The foci development and counting were performed as previously described [13]. The limit of the detection is 1:20.

### 2.4. Statistical Analysis

Categorical variables were analysed using the chi-square test. Geometric mean antibody titres with 95% confidence intervals (CIs) were calculated, and neutralizing activities were expressed as medians and interquartile ranges (IQRs). Age and Sex adjustments were performed. We performed the one-way ANCOVA with Bonferroni correction to assess the differences of antibody titer. The neutralizing activity was evaluated using the Kruskal–Wallis method with Dunn’s correction using SPSS v23.0 (IBM Corp, Armonk, NY, USA). Figures were generated using GraphPad Prism v9.0 (GraphPad, San Diego, CA, USA). A *p*-value < 0.05 was considered statistically significant.

## 3. Results

### 3.1. Demographic Data

The group who had been vaccinated with two doses of CoronaVac and subsequently became infected with SARS-CoV-2 contained 77 patients, with a mean age of 34.2 years; this group was comprised of 58 women (75.3%) and 19 men (24.7%). The median time between the second CoronaVac vaccination and breakthrough infection was 88 days (IQR 68–100 days). Blood samples were collected at 34 days (IQR 29–43) post infection. The group who had been fully vaccinated with CoronaVac but remained unexposed to SARS-CoV-2 infection contained 170 participants, with a mean age of 42.3 years; this group was comprised of 89 women (52.4%) and 81 men (47.6%). The blood samples were collected at a median of 29 days (IQR 27–31), following second dose vaccination. The group who had been fully vaccinated with CoronaVac, then received AZD1222 as a third vaccination, contained 210 participants, with a mean age of 40 years; this group was comprised of 151 women (71.9%) and 59 men (28.1%). The median time between blood sampling and a third dose was 28 days (IQR 20–32) (Table 1).

### 3.2. Binding Antibody Measurement

**Figure 1:**
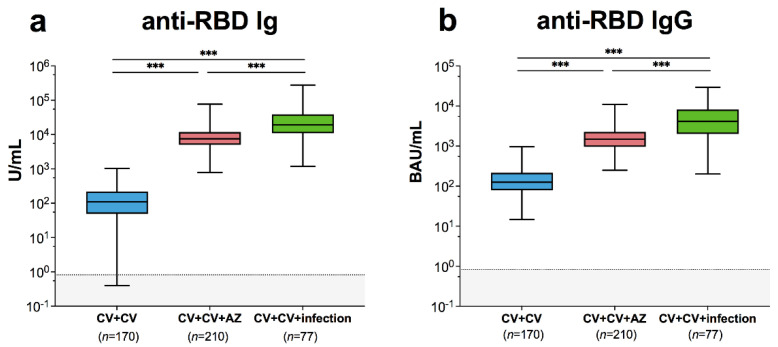
SARS-CoV-2-specific binding antibody responses and neutralizing activities. Immune response of individuals with SARS-CoV-2 breakthrough infection (CV + CV + Infection) was compared to those with fully vaccinated CoronaVac vaccines without infection (CV + CV) and those who received AZD1222 as third booster (CV + CV + AZ). Serum (**a**) anti-RBD Ig activity, (**b**) anti-RBD IgG-binding antibody units. The results are shown as Geometric mean titres with 95% CIs. The cut-off value is indicated as dotted lines. Statistics were calculated using one-way ANOVA with Bonferroni correction. *** *p* < 0.001.

Total RBD-specific Ig was significantly increased in patients with breakthrough infection (19698 units per millilitre (U/mL); 95% CI 15335–25302 U/mL) compared to fully vaccinated individuals without infection (98 U/mL; 95% CI 83–116 U/mL) (Figure 1a). RBD-specific Ig was higher in participants with breakthrough infection than in those who received AZD1222 as a third vaccination (7947 U/mL; 95% CI 7277–8679). Anti-RBD IgG was significantly higher in patients with breakthrough infection (3946 binding antibody units per millilitre (BAU)/mL; 95% CI 3135–4965 BAU/mL) compared to fully vaccinated individuals (128 BAU/mL, 95% CI 114–144 BAU/mL) and those who received AZD1222 as a third vaccination (1492 BAU/mL, 95% CI 1367–1629 BAU/mL) (Figure 1b). These results indicate that patients with breakthrough infection could mount SARS-CoV-2-specific binding antibody responses.

### 3.3. Neutralization Assay

**Figure 2:**
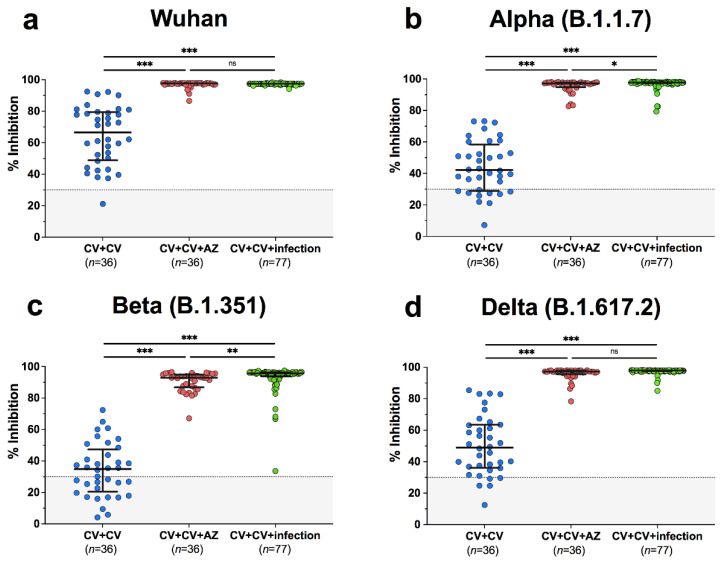
Neutralizing activities against (**a**) wild-type, (**b**) B.1.1.7-alpha, (**c**) B.1.351-beta, and (**d**) B.1.617.2-delta using surrogate virus neutralization test (sVNT). The fully vaccinated individuals with two doses of CoronaVac (CV+CV), the fully vaccinated individuals with two doses of CoronaVac then administered a third vaccination with AZD1222 (CV+CV+AZ), and the fully vaccinated individuals with two doses of CoronaVac followed by SARS-CoV-2 breakthrough infection (CV+CV+infection) were compared. Median values with IQRs are shown as horizontal bars. Dotted lines indicate cut-off values, and grey shaded areas depict values under the cut-off. Statistics were calculated using Kruskal–Wallis tests with Dunn’s post hoc correction. * *p* < 0.05, ** *p* < 0.01, *** *p* < 0.001. ns indicates no statistical significance.

**Figure 3:**
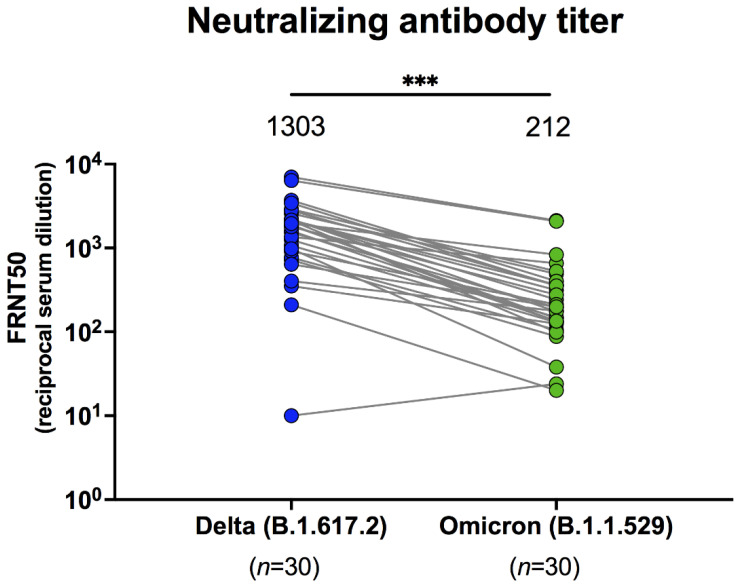
Live SARS-CoV-2 serum dilution titres were determined against B.1.617.2-delta and B.1.1.529-omicron in serum samples from individuals with breakthrough infection determined using focus reduction neutralization test 50 values (FRNT50). Number indicates the geometric mean titres with 95% CIs. Statistics were calculated using Wilcoxon matched pair signed rank test. *** *p* < 0.001.

Among breakthrough infection patients, 77/77 (100%) exhibited potential neutralization, determined using sVNT against wild-type and SARS-CoV-2 variants. The median neutralizing activities in patients with breakthrough infections were 97.5% for wild-type, 97.7% for alpha, 95.6% for beta, and 97.9% for delta (Supplementary Table S1). Neutralizing activities in breakthrough infection patients were significantly higher than those in unexposed individuals, following complete CoronaVac vaccination (*p* < 0.001) (Figure 2a–d). Compared to individuals who received AZD1222 as a third vaccination, the number of patients with breakthrough infection neutralizing activity was significantly higher for alpha (*p* = 0.02) and beta (*p* < 0.01) variants, but not wild-type or delta variants. This suggests that variant cross-neutralization was improved after breakthrough infection. With respect to neutralizing antibody titres in sera from patients with breakthrough infection, determined using a live virus neutralization test, focus reduction neutralization test (FRNT50), the geometric mean titres against delta were 1303 (95% CI 830–2048), whereas those against omicron were 212 (95% CI 142–316) (Figure 3). This indicates that omicron is less effectively neutralized by serum antibodies derived from breakthrough infection, with a 6.2-fold reduction compared to delta (*p* < 0.001).

## 4. Discussion

Our findings indicate that patients with breakthrough infection exhibited potent antibody responses, with titres exceeding those of individuals who received AZD1222 as a third vaccination. Notably, the levels of anti-RBD IgG in almost all breakthrough patients were higher than 506 BAU/mL, which was the antibody level associated with 80% effectiveness against symptomatic alpha variant infection in a previous report [14]. Breakthrough infection increased neutralizing activity that cross-inhibited SARS-CoV-2 variants, corresponding to wild-type, alpha, beta, and delta. In a previous study, a similar result was observed in individuals who had received two mRNA SARS-CoV-2 vaccinations and subsequently became infected [15]. This indicates that combined natural immunity and vaccine-induced immunity results in higher neutralizing potency than vaccination or infection alone [16].

Omicron variants are reportedly less susceptible to neutralization by existing immunity after natural infection [17], and vaccine-induced immunity [18]. Notably, our previous study reported the neutralizing antibody titres against delta and omicron, following two doses of CoronaVac, were less than the limit of detection (< 20) [11], while the neutralizing antibody titres measured by serum from individuals who received AZD1222 as the third dose were 1003 and 250 against delta and omicron, respectively. The neutralizing antibody titres against delta and omicron between the AZD1222 third booster from our previous study and breakthrough infection in the current study were comparable. However, sera from patients with breakthrough infection in the present study also exhibited less neutralizing activity against omicron than against delta. Consistent with a previous study [8], neutralization against omicron was 11.7-fold less than that for wild-type, and 7.9-fold less than that for delta. These results suggest immune escape by omicron in breakthrough infection patients. Reduced neutralizing potency against the omicron variant may be associated with many mutations in the spike protein [19].

The limitations of this study include the small sample size, and the SARS-CoV-2 variants that caused breakthrough infections were not identified. Omicron neutralization assays were not conducted using sera from unexposed individuals who had been fully vaccinated with two doses of CoronaVac, or those who received AZD1222 as a third vaccination. Further investigations are needed to overcome the limitations.

## 5. Conclusions

Our findings highlight that breakthrough infection after two doses of inactivated vaccines can induce neutralizing antibodies against omicron. Further investigation is needed to assess the long-term persistence of antibodies against the omicron variant. This scientific knowledge of the immune response against new-emerging SARS-CoV-2 variants may support the implication of vaccine planning and the development of matching public health strategies.

## Figures and Tables

**Table 1:** Characteristics of participants in the study.

Characteristics	CV + CV (*n* = 170)	CV + CV + AZ (*n* = 210)	CV + CV + INF (*n* = 77)	*p*-Value
Sex (*n*, %)				
female	89 (52.4%)	151 (71.9%)	58 (75.3%)	*p* < 0.001 ^a^, *p* < 0.001 ^b^, *p* = 0.56 ^c^
male	81 (47.6%)	59 (28.1%)	19 (24.7%)	
Age in years (mean, SD)	42.3 (9.6)	40.0 (9.8)	34.2 (9.9)	*p* = 0.02 ^d^, *p* < 0.001 ^e^, *p* < 0.001 ^f^
Interval between 1st and 2nd dose (median, IQR)	23 (21–26)	21 (21–26)	23 (21–28)	
Interval between 2nd and 3rd dose (median, IQR)	N/A	70 (61–79)	N/A	
Interval between 2nd dose and symptom onset (median, IQR)	N/A	N/A	88 (68–100)	
Interval between last vaccination and blood sampling (median, IQR)	29 (27–31)	28 (20–32)	N/A	
Interval between symptom onset and blood sampling (median, IQR)	N/A	N/A	34 (29–43)	

CV + CV, fully vaccinated with two doses of CoronaVac; CV + CV + AZ, fully vaccinated with two doses of CoronaVac then administered a third vaccination with AZD1222; CV + CV + INF, fully vaccinated with two doses of CoronaVac followed by SARS-CoV-2 breakthrough infection; IQR or interquartile range refers to the number of days between the events; N/A, no data available; SD, standard deviation. ^a,b,c^ represent the comparison between CV + CV vs CV + CV + AZ, CV + CV vs CV + CV + INF and CV + CV + AZ vs CV + CV + INF, respectively, using Chi-square test. ^d,e,f^ represent the comparison between CV + CV vs CV + CV + AZ, CV + CV vs CV + CV + INF, CV + CV + AZ vs CV + CV + INF, respectively, using *t*-test.

## Data Availability

All data are available in the report. Additional information can be requested from the corresponding author.

## Supplementary Materials

The following supporting information can be downloaded at: https://www.mdpi.com/article/10.3390/vaccines10030391/s1: Figure S1: Percentage of SARS-CoV-2 variants circulating in Thailand between 21 April 2021 and 20 September 2021. Table S1: Binding antibody levels including total anti-RBD Ig and anti-RBD IgG. Neutralizing activities of sera from the three groups were measured via surrogate virus neutralization tests.
