# Supplementary material for: COVID-19 Breakthrough Infection after Inactivated Vaccine Induced Robust Antibody Responses and Cross-Neutralization of SARS-CoV-2 Variants, but Less Immunity against Omicron
Source: Vaccines (Basel). 2022 Mar 3;10(3):391. doi: 10.3390/vaccines10030391 (PMC8949546; doi:10.3390/vaccines10030391)
Supplement: Supplementary file 1 [file vaccines-10-00391-s001.zip › vaccines-1619507-supplementary.pdf]

Supplementary Information

Figure S1. Percentage of SARS-CoV-2 variants circulating in Thailand between 21 April 2021 and 20 September 2021.

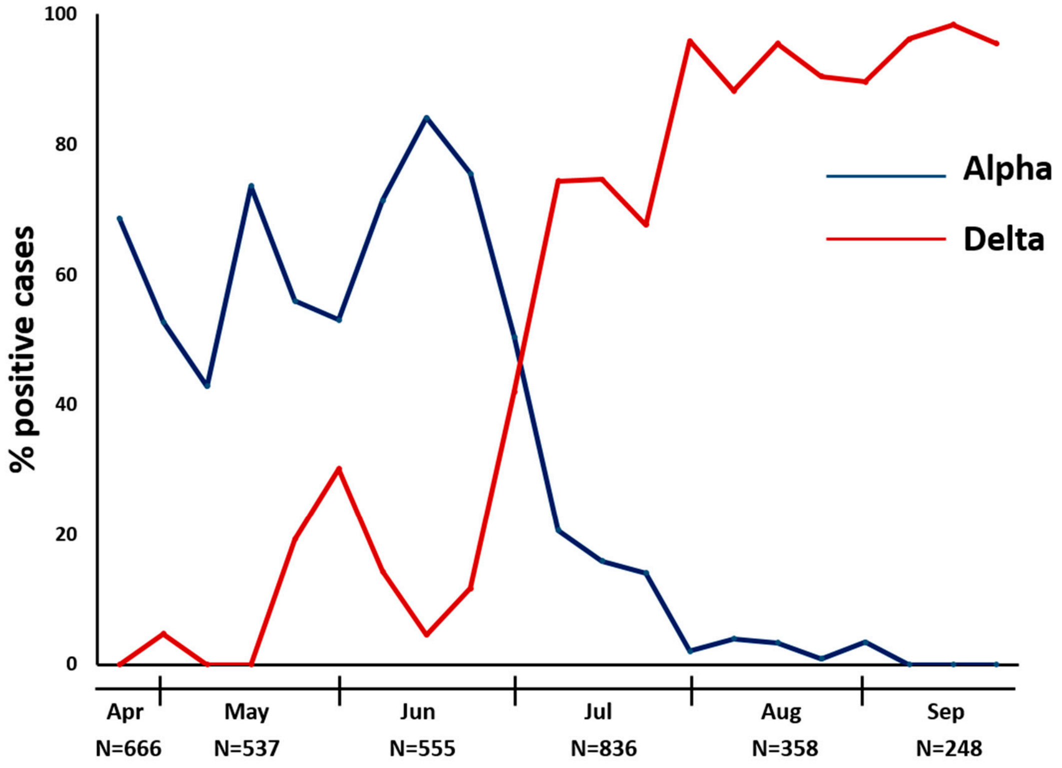

Table S1. Binding antibody levels including total anti-RBD Ig and anti-RBD IgG. Neutralizing activities of sera from the three groups were measured via surrogate virus neutralization tests.

	**CV+CV (*n* = 170)**	**CV+CV+AZ (*n* = 210)**	**CV+CV+INF (*n* = 77)**
Anti-RBD-Ig			
n	170	210	77
GMT (95% CI)	98 (83–116)	7947 (7277–8679)	19,698 (15335–25,302)
Anti-RBD IgG			
n	170	210	77
GMT (95% CI)	128 (114–144)	1492 (1367–1629)	3946 (3135–4965)
sVNT-wuhan			
n	36	36	77
Median (IQR)	66.6 (48.9–79.4)	97.7 (97.0–97.8)	97.5 (97.3–97.6)

sVNT-B.1.1.7 (alpha)<br>*n*<br>Median (IQR)	36<br>42.1 (29.0–58.3)	36<br>97.2 (94.7–97.7)	78<br>97.7 (97.3–98.0)
sVNT-B.1.351 (beta)<br>*n*<br>Median (IQR)	36<br>34.8 (20.5–47.3)	36<br>92.9 (86.8–94.8)	78<br>95.6 (93.9–96.2)
sVNT-B.1.617.2 (delta)<br>*n*<br>Median (IQR)	36<br>48.9 (36.1–63.4)	36<br>97.2 (95.6–97.9)	78<br>97.9 (97.7–98.0)

CI, confidence interval; CV+CV, fully vaccinated with two doses of CoronaVac; CV+CV+AZ, fully vaccinated with two doses of CoronaVac then administered a third vaccination with AZD1222; CV+CV+INF, fully vaccinated with two doses of CoronaVac followed by SARS-CoV-2 breakthrough infection; GMT, geometric mean titre; IQR, interquartile range; RBD, receptor-binding domain
